# Safety, Immunogenicity, and Protective Efficacy of a Chimeric A/B Live Attenuated Influenza Vaccine in a Mouse Model

**DOI:** 10.3390/microorganisms9020259

**Published:** 2021-01-27

**Authors:** Ekaterina Stepanova, Elena Krutikova, Pei-Fong Wong, Victoria Matyushenko, Ekaterina Bazhenova, Irina Isakova-Sivak, Larisa Rudenko

**Affiliations:** Institute of Experimental Medicine, 197376 Saint Petersburg, Russia; krutikova.iem@mail.ru (E.K.); po333222@gmail.com (P.-F.W.); matyshenko@iemspb.ru (V.M.); sonya.01.08@mail.ru (E.B.); isakova.sivak@iemspb.ru (I.I.-S.); vaccine@mail.ru (L.R.)

**Keywords:** live attenuated influenza vaccine, LAIV, influenza virus, genome packaging, reassortment, influenza B virus, universal influenza vaccine, chimeric hemagglutinin

## Abstract

Influenza A and B viruses cause significant morbidity and mortality worldwide. Current influenza vaccines are composed of three or four strains: A/H1N1, A/H3N2, and B (Victoria and Yamagata lineages). It is of great interest if immunization against both type A and B influenza viruses can be combined in a single vaccine strain, thus reducing the cost of vaccine production and the possibility of strain interference within the multicomponent vaccine. In the current study, we developed an experimental live cold-adapted influenza intertype reassortant (influenza A and B) vaccine on the live attenuated influenza vaccine (LAIV) A/Leningrad/134/17/57 backbone. Hemagglutinin (HA) and neuraminidase (NA) functional domains were inherited from the influenza B/Brisbane/60/2008 strain, whereas their packaging signals were substituted with appropriate fragments of influenza A virus genes. The recombinant A/B virus efficiently replicated in eggs and Madin–Darby Canine Kidney (MDCK) cells under optimal conditions, temperature-sensitive phenotype was maintained, and its antigenic properties matched the influenza B parental virus. The chimeric vaccine was attenuated in mice: after intranasal immunization, viral replication was seen only in nasal turbinates but not in the lungs. Immunological studies demonstrated the induction of IgG antibody responses against the influenza A and B virus, whereas hemagglutination inhibition (HAI) and neutralizing antibodies were detected only against the influenza B virus, resulting in significant protection of immunized animals against influenza B virus challenge. IFNγ-secreting CD8 effector memory T cells (CD44^+^CD62L^−^) were detected in mouse splenocytes after stimulation with the specific influenza A peptide (NP_366_); however, the T-cell response was not sufficient to protect animals against infection with a high-dose mouse-adapted A/California/07/2009 (H1N1pdm09) virus, most probably due to the mismatch of key T-cell epitopes of the H1N1 virus and the LAIV backbone. Overall, generation of the chimeric A/B LAIV virus on a licensed LAIV backbone demonstrated prospects for the development of safe and efficacious vaccine candidates that afford combined protection against both type A and type B influenza viruses; however, further optimization of the T-cell epitope content within the LAIV backbone may be required.

## 1. Introduction

Seasonal influenza vaccines contain influenza A and B viruses, providing optimal protection against circulating influenza strains. The need for influenza vaccines with broad protection is urgent to reduce the influenza burden worldwide. Live attenuated influenza vaccines (LAIVs) provide broader cross-protection compared to inactivated vaccines due to the considerable involvement of T-cell immunity, the generation of resident memory T cells targeted at conserved viral epitopes in respiratory airways, and the induction of mucosal secretory antibody responses [1,2,3,4].

Influenza B viruses are less variable than influenza A viruses [5,6]. Two lineages of influenza B are specified on the basis of hemagglutinin (HA) antigenic properties: sera against one lineage do not cross-react with viruses of another lineage [6], although some level of cross-protection has been demonstrated [7,8]. The highest burden of influenza B virus infection is registered in children, but these viruses can also cause complicated cases in the elderly population [9,10]. Natural reassortment between the influenza A and B viruses does not occur, which is restricted by the specificity of viral RNA packaging signals [11,12]. For effective replication of a cross-type reassortant, specific features of the polymerase have to match the features of the virus subtype, first of all, the structure of promoter elements in the noncoding regions of RNA segments. The impact of other viral regions on polymerase machinery activity has also been shown [13]. Nevertheless, the capacity of the influenza genome to express foreign proteins has been extensively studied in multiple experiments with reporter genes and chimeric constructs [14,15,16,17,18,19]. These studies have shown which gene fragments are necessary for packaging genome segments into virions [14,16]. Engineered reassortants between the influenza A and B viruses have been previously obtained by several study groups using a H1N1 influenza virus backbone [11,12,20,21]. Horimoto et al. [20] studied different variants of A/B chimeric HAs on the base of the A/WSN/33 strain with the insertion of B/Lee/40 fragments. In Flandorfer et al. [12], the reassortants with both HA and neuraminidase (NA) were rescued on the base of the A/WSN/33 and B/Yamagata/16/88 strains. The same influenza B strain was used by Baker et al. [11]; as a backbone, A/PR/8/34 virus was used. Some of the chimeric constructions were tested for biological characteristics and immunogenicity in animal models [21,22]. In a study by Horimoto et al. [22], immunogenicity of the chimeric A/B virus was investigated by an hemagglutination inhibition (HAI) assay with influenza B antigen. For live viral vaccines, it was established that one of the major mechanisms providing protection against respiratory viruses is the formation of resident memory T-cell responses in the respiratory airways, targeted at epitopes of different pathogen’s proteins [23]. These epitopes may arise from the fragments of conservative protein domains, providing basic protection against different virus subtypes in the future. Therefore, experimental assessment of immunogenicity and protective potential of a chimeric live vaccine based on influenza A and B reassortants can be informative for the development of universal cross-type influenza vaccines.

The A/Leningrad/134/17/57 (H2N2) strain is licensed for influenza A LAIV strain development [24]. Since this LAIV backbone virus has a strong safety record and has been on the market for decades, its use for designing a new generation of influenza vaccines is a very promising strategy. All previously studied chimeric constructions were obtained on the base of H1N1 strains. However, those viruses cannot be widely used for the development of human live attenuated vaccines due to the lack of safety data. We aimed to study if the approach of engineering intertype reassortants can be applied to the cold-adapted attenuated influenza A virus of the H2N2 subtype.

In this study, we rescued an engineered influenza A/B reassortant on the backbone of a licensed Russian LAIV master donor virus—the strain A/Leningrad/134/17/57 (H2N2)—and assessed its biological characteristics, stability, immunogenicity, and protective efficacy in a mouse model.

## 2. Materials and Methods

### 2.1. Viruses and Plasmids

A/Leningrad/134/17/57 (H2N2) (A/Len/17) is a cold-adapted master donor virus (MDV) for a Russian LAIV and was used as a backbone for the chimeric vaccine strain (sequences are available in the GISAID database under isolate identifier EPI_ISL_169836). HA (EPI753679) and NA (EPI902445) gene segments were inherited from the B/Brisbane/60/2008 (B/Victoria) strain with modifications in the genome packaging signals. Two classical LAIV reassortants were used as control vaccines in mouse experiments: B/60/Brisbane/2008/83 (B/LAIV) (B/Brisbane/60/2008-based LAIV on Russian MDV B/USSR/60/69 backbone) and A/17/New York/2015/5364 (A/LAIV) (A/New York/61/2015-based H1N1pdm09 LAIV on A/Len/17 backbone). All vaccine viruses and wild-type A/Leningrad/134/57 (H2N2) and B/Brisbane/60/2008 viruses were obtained from the influenza virus repository of the Institute of Experimental Medicine. A mouse-adapted A/California/07/2009 (H1N1pdm09) strain was obtained from the influenza virus repository of the Smorodintsev Research Institute of Influenza (St. Petersburg, Russia). The latter strain was derived after eight sequential passages of the original egg-grown A/California/07/2009 virus in the lungs of BALB/c mice, followed by cloning of the virus mixture by limiting dilutions in eggs.

A chimeric A/B LAIV virus (H2B) was rescued as previously described [24] by electroporation of Vero cells with a set of 8 plasmids (6 plasmids encoding the PB1, PB2, PA, NP, M, and NS genes of A/Len/17, and the chimeric A/B HA, and NA genes). Electroporation was performed using the Neon Transfection system (Invitrogen, Life Technologies Corporation, Carlsbad, CA, USA) according to the manufacturer’s instructions.

Chimeric NA was constructed through PCR amplification with specific primers to replace untranslated regions (UTR) of type B NA to appropriate regions of Len/17 NA (F primer 5′-AGCAAAAGCAGGAGTGAAAATGCTACCTTCAACTATAC-3′; R primer 5′-AGTAGAAACAAGGAGTTTTTTCTAAAATTGCGAAAGC TTACAGAGCCATGTC-3′). Chimeric HA was constructed from three fragments amplified with specific primers containing additional BsmBI sites; a chimeric segment was then combined through BsmBI digestion and T4 ligation (for HA 3′ UTR and signal peptide: F primer 5′-GATCGCTCTTCAGGGAGCAAAAGCAGGGG-3′; R-primer 5′-TATCGTCTCTGATCCCCTCTCACTGCTGTGAACAG-3′; for influenza B ectodomain amplification: F primer 5′-TATCGTCTCAGATCGAATCTGCACTGG-3′; R primer 5′-CGTCGTCTCGATTATCCAATCCATC-3′; and for the HA fragment with transmembrane domain (TMD) and cytoplasmic domain (CPD): F primer 5′-GCTCGTCTCCTAATTATCAAATCCTTGCC-3′, R primer 5′-ACTGGCTCTTCTATTAGTAGAAACAAGGGTGTTTT-3′).

### 2.2. In Vitro Studies

#### 2.2.1. Growth Characteristics in Eggs, ts/ca Phenotype

Viruses were propagated in chicken embryos 10–11 days old at 33 °C. After 72 h incubation, allantoic fluid was harvested, clarified by low-speed centrifugation, and stored in aliquots at −70 °C. For *ts* (temperature-sensitive) and *ca* (cold-adapted) phenotype determination, a viral titer at optimal incubation temperature (33 °C) was compared to a titer at high (37 °C, 38 °C, and 39 °C) and low (26 °C) temperatures, respectively. For titers at 33–39 °C, the infected eggs were incubated for 72 h; for titers at 26 °C, the eggs were incubated for 7 days. The virus was detected by hemagglutination (HA) assay using 1% chicken red blood cells (RBC) [25], and the titers were calculated by the Reed and Muench method [26] and expressed as lg EID_50_/mL (50% egg infective dose).

#### 2.2.2. Growth Kinetics in MDCK Cells

Viral titers in Madin–Darby Canine Kidney (MDCK) cells were detected both by an HA assay and by a cell-based enzyme-linked immunosorbent assay (ELISA) using a horseradish peroxidase (HRP)-conjugated monoclonal antibody to influenza A nucleoprotein (NP). For growth dynamics in the MDCK study, 96-well plates with a MDCK cell monolayer were inoculated with 10-fold virus dilutions in a volume of 25 μL. The supernatants were tested for the presence of influenza virus by the HA assay at 24, 48, 72, and 96 h.p.i. (hours post infection). The residual medium was then removed, and the cell monolayer was fixed with ice-cold 80% acetone for 20 min at −20 °C. After washing with PBS-T (PBS containing 0.05% Tween-20), plates were incubated with 5% non-fat milk (PZN 460553, Spinnrad GmbH, Bad Segeberg, Germany) for 1 h at 37 °C to block nonspecific binding. After removal of the blocking buffer, the plates were incubated with 50 μL/well of 3% hydrogen peroxide for 20 min at room temperature to inactivate endogenous peroxidase and then washed with PBS-T. After washing, cells were incubated with an HRP-conjugated monoclonal antibody to influenza NP for 1 h at 37 °C. After washing with PBS-T, a one-step ultra-tetramethylbenzidine (TMB) substrate solution (Thermofisher Scientific, Waltham, MA, USA, cat#34028) was added, and the plates were incubated until the blue color was developed. The reaction was stopped with 25 μL of 2n H_2_SO_4_, and optical density (OD) was measured at 450 nm using a Bio-Rad Xmark Microplate reader (Bio-Rad, Hercules, CA, USA). The viral titer was calculated using the Reed and Muench method, where the wells were considered positive if the OD_450_ value exceeded the OD_450_ of the control cells by at least twofold.

#### 2.2.3. Study of Antigenic Properties

Antigenic properties of the chimeric A/B virus were studied by the HAI assay by a standard protocol [25]. For HAI, hyperimmune rat antisera to B/Brisbane/60/2008 (B/Victoria), A/Leningrad/134/17/57 (A/H2N2), B/Phuket/3073/2013 (B/Yamagata), A/Brisbane/02/2018 (A/H1N1pdm09), and A/Switzerland/8060/2017 (A/H3N2) were used.

#### 2.2.4. HA Thermostability

The thermostability of HA was studied after virus heating in the range of temperatures 37–70 °C. Chorioallantoic fluid with the virus was diluted 1:5 with PBS and then heated to a distinct temperature for 20 min. After this treatment, hemagglutination with 1% chicken RBC was assessed in a standard test with 2-fold dilutions of the virus.

#### 2.2.5. Genetic Stability

The genetic stability of the chimeric virus was studied by full and partial sequencing after passaging in eggs. Full sequencing of the virus stock was performed. RNA was purified from chorioallantoic fluid with a QIAamp Viral RNA Mini Kit (QIAGEN, MD, USA, cat#52904). For reverse transcription and PCR, a Biolabmix Biomaster RT-PCR kit was used (Biolabmix, Novosibirsk, Russia) with specific primers. The purification of PCR products was conducted with a Clean-Up mini kit (Evrogen, Moscow, Russia). Sequencing was performed with an ABI Prism 3031xl Genetic Analyzer (Applied Biosystems, Waltham, MA, USA) with a BrilliantDye™ Terminator, v 3.1 (NimaGen, Nijmengen, The Netherlands, cat#BRD3-100) according to the manufacturer’s manual.

The stability of attenuating mutations, specific for A/Len/17 [24], was studied after 10 sequential passages in chicken embryos by partial sequencing with the PyroMark Q24 System (QIAGEN, MD, USA). RNA purification and RT-PCR were performed with the same reagent kits. Purification of the PCR products with DNA chain separation was performed with appropriate buffers (QIAGEN, MD, USA) and Sepharose High Performance (GE Healthcare, Chicago, IL, USA) according to the PyroMark Q24 manual. Pyrosequencing reaction was performed with PyroMark Q24 Gold Reagents (QIAGEN, MD, USA).

### 2.3. Animals

Six- to eight-week-old female C57BL/6J mice were purchased from the Stolbovaya breeding laboratory and nursery of the Scientific Center for Biomedical Technologies of the Federal Medical and Biological Agency (Moscow region, Russia).

### 2.4. Ethics Statement

All animal experiments and manipulations were performed according to Directive 2010/63/EU of the European parliament and of the council of 22 September 2010 on the protection of animals used for scientific purposes [27] and with the approval of the Institute of Experimental Medicine Ethics Committee (ethical approval number #1/20 dated 27 February 2020).

### 2.5. Mouse Study Design

**Figure 1:**
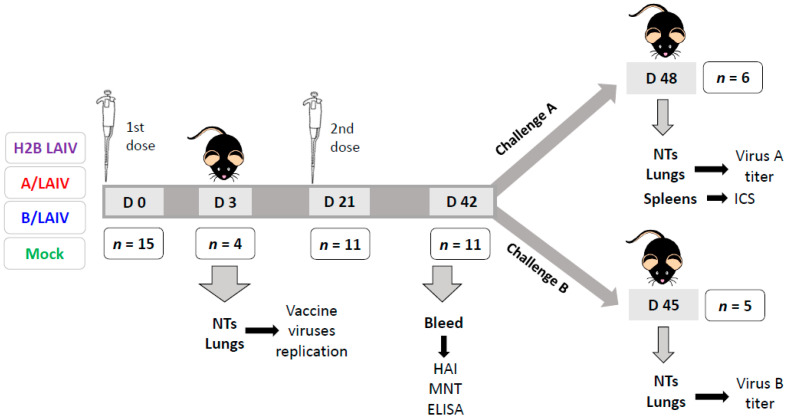
Experiment design scheme: the mice were twice immunized with experimental chimeric (H2B) vaccine or control live attenuated influenza vaccines (LAIVs). On day 3 after immunization, vaccine virus titers in the organs were assessed in 4 animals from each group. Three weeks after the second immunization, the animals were challenged with influenza A or B virus. Five animals stayed naïve for negative controls. Viral loads in the lungs were studied on day 3 (for influenza B) and day 5 (for influenza A) after the challenge. The number of animals in each group is indicated in frames.

Mice were intranasally immunized with the chimeric A/B virus or control LAIV viruses at a dose of 6.0 lgEID_50_ in 50 μL, twice, with a 21-day interval. For the mock-immunized group, PBS was used for inoculation in the same volume. On day 3 of the experiment, replication of the vaccine viruses in nasal turbinates and lungs was assessed by titration of tissue homogenates in chicken embryos as described in Section 2.7. For protection assessment, mice were challenged with the influenza A or influenza B viruses on day 21 after the second immunization. For B/Brisbane/60/2008, the challenge dose was 6.0 lgEID_50_/50 μL; for the mouse-adapted A/California/07/2009, the challenge dose was 3.0 lgEID_50_/50 μL. Protection was monitored by viral load in the lungs at 3 dpi for influenza B virus and 5 dpi for influenza A virus challenge. The scheme of the experiment is illustrated in Figure 1.

### 2.6. Immunogenicity Studies

Serum antibody responses were studied via HAI, ELISA, and a micro-neutralization test (MNT) 3 weeks after the second vaccine dose. Three antigens were used for these tests: B/Brisbane/60/2008, the H2B chimeric virus, and A/17/New York/2015/5364 (H1N1pdm09). The reason for using a reassortant A/Len/17-based A/LAIV strain as an antigen rather than the wild-type influenza A virus is so that the possible induction of antibodies to viral proteins other than HA and NA could be detected. T-cell immunity was studied by assessing the levels of virus-specific effector memory (CD44^+^CD62L^−^) cytotoxic T lymphocytes (CTL) in mouse splenocytes five days after influenza A virus challenge (Figure 1).

#### 2.6.1. Hemagglutination Inhibiting Antibody

For HAI assay, serum samples were treated by the receptor-destroying enzyme (RDE II, Denka Seiken, Tokyo, Japan) overnight at 37 °C, followed by incubation at 56 °C for 1 h to inactivate neuraminidase activity. The HAI assay was performed by a standard protocol [25], with twofold serum dilutions starting from 1:10, using 1% chicken RBCs.

#### 2.6.2. Serum IgG Antibody

For ELISA, viruses were purified on a sucrose density gradient as described elsewhere [28]; 96-well high-sorbent microplates (Corning, NY, USA, cat#CLS2592) were coated with 16 hemagglutinating units of appropriate virus in a volume of 50 μL per well. After washing with PBS-T, plates were incubated with 1% bovine serum albumin in PBS for 1 h at 37 °C to block nonspecific binding. Twofold serum dilutions in PBS were prepared starting from 1:20, and sera were incubated in plates for 1 h at 37 °C. After washing, incubation with Goat Anti-Mouse IgG H&L (HRP) (ab205719) was performed for 1 h at 37 °C. After washing with PBS-T, the TMB substrate solution was added, and after the blue color was developed, the reaction was stopped with 25 μL of 2n H_2_SO_4_. Optical density (OD) was measured at 450 nm. The IgG antibody titer was assigned as the last dilution with OD_450_ exceeding the OD_450_ of control wells at least twofold without the addition of serum.

#### 2.6.3. Virus Neutralizing Antibody

The microneutralization test (MNT) was performed in 96-well cell culture plates seeded with MDCK cells 24 h prior to the infection. The 75–95% confluent monolayers were inoculated with twofold serum dilutions prepared on DMEM culture medium (DMEM high glucose (4.5 g/L), with L-glutamine (Capricorn Scientific, Ebsdorfergrund, Germany), with antibiotic-antimycotic (Gibco, Thermofisher scientific, Waltham, MA, USA) mixed 1:1 with the virus at a dose of 100 TCID_50_ (50% tissue infective doses). Plates were incubated at 37 °C, 5% CO_2_ for 48 h, and the virus was then detected by cell-based ELISA as described above (Section 2.2.2). The MNT titer was calculated as the last dilution with OD_450_, lower than the cutoff value calculated as follows:

average OD_450_ of cell control + (average OD_450_ of virus control − average OD_450_ of cell control)/2.

#### 2.6.4. T-Cell Immune Responses

To assess T-cell responses to influenza A virus, an intracellular cytokine staining (ICS) assay was performed as previously described [29]. Briefly, splenocytes were isolated 5 days after the influenza A virus challenge according to the design of the experiment described in Section 2.5. The cells were resuspended in an RPMI-1640 medium supplemented with 10 mM HEPES buffer (Gibco, Thermofisher scientific, Waltham, MA, USA), 20 U/mL IL-2, GlutaMAX (Gibco, Thermofisher scientific, Waltham, MA, USA, cat#35050061), antibiotic-antimycotic (Gibco, Thermofisher scientific, Waltham, MA, USA, cat#15240062), 50 μM β-mercaptoethanol, and 10% FBS (Gibco, Thermofisher scientific, Waltham, MA, USA) and adjusted to a concentration of 10^7^ cells/mL. One million cells were used for stimulation either with 1 µg of influenza NP_366-374_ (ASNENMDTM (IEDB ID 4580)) peptide or with 50 ng/mL phorbol myristate acetate (PMA) as a positive control. Non-stimulated cells were used to assess background cytokine production. Brefeldin A (Biolegend, San Diego, CA, USA, cat#420601) was added to each well for 5 h, followed by sequential staining with surface antibody anti-CD4 (RM4-5), anti-CD8 (53–6.7), anti-CD62L (MEL-14), anti-CD44 (IM7), and live/dead fixable stain ZombieAqua (BioLegend, San Diego, CA, USA); fixation/permeabilization with Cytofix/Cytoperm (BD Biosciences, San Jose, CA, USA, cat#554715); and finally staining with the intracellular cytokine antibody anti-INFγ (XMG1.2) (Biolegend, San Diego, CA, USA). Samples were fixed in 1% formaldehyde and further analyzed on a Navios flow cytometer (Beckman Coulter, Brea, CA, USA). Data analysis was performed using the FlowJo software (TriStar).

### 2.7. Viruses Replication in Lung Tissue and Nasal Turbinates

For virus titer assessment, the organs were homogenized in PBS (1 mL per organ) using a small bead mill TissueLyser LT (QIAGEN, MD, USA) for 10 min and then centrifuged for 2 min at 6000 rpm at 4 °C. The supernatants were titrated by limiting dilutions in embryonated chicken eggs. Virus titers were assessed after incubation for 3 days at 33 °C by virus detection in an HA assay using 1% chicken RBCs.

### 2.8. Statistical Analysis and Data Visualization

All calculations of viral loads were performed with log_10_ load values, and calculations with antibody titers were performed for log_2_ titer values. Normality was assessed with the D’Agostino and Pearson or Shapiro–Wilk normality test. Differences between groups were calculated with an ordinary one-way ANOVA or Kruskal–Wallis test with appropriate multiple comparisons tests (Dunn’s, Dunnet’s, and Tukey’s). Tests and calculated *p* values are indicated on the figures and in figure captions. Differences with *p* ≤ 0.05 are considered significant. GraphPad Prism 7 was used for calculations and figure preparation.

## 3. Results

### 3.1. H2B Chimera Design

**Figure 2:**
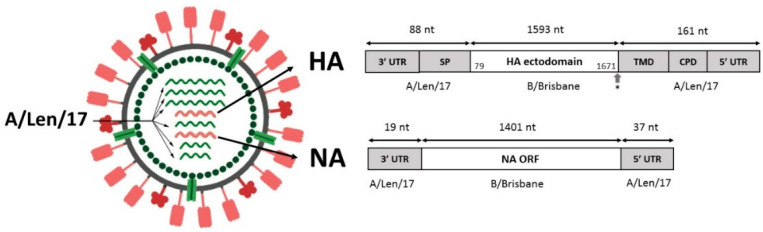
Chimeric A/B genome composition and a scheme of hemagglutinin (HA) and neuraminidase (NA) genetic segments: the chimeric H2B reassortant virus contains six genetic segments from A/Leningrad/134/17/57 (A/Len/17) (H2N2) LAIV master donor virus (MDV) (shown in green). Segments encoding HA and NA are engineered constructs on the base of parts of A/Len/17 and B/Brisbane/60/2008 (B/Brisbane) (shown in red). In the scheme of segment constructions, exact numbers of nucleotides are specified. For the B/Brisbane HA ectodomain, the positions of start and end nucleotides in the B/Brisbane/60/08 HA segment are indicated. At the end of the B/HA ectodomain, His545 was changed to Tyr, corresponding to A/Len/17 appropriate position (shown with grey arrow). SP: HA signal peptide, TMD: transmembrane domain, CPD: cytoplasmic domain, UTR: untranslated region.

For the purpose of designing a chimeric influenza A/B virus, we reviewed successful strategies published in the literature [11,12,18,19,20]. In the final design, our chimeric virus was a reassortant that inherited six genes encoding internal and nonstructural genes from A/Len/17 MDV, whereas HA and NA segments were chimeric (Figure 2). For the NA segment, only UTRs of the influenza B virus gene were substituted with the corresponding regions of A/Len/17 NA. For the HA segment, the ectodomain was transferred from influenza B virus, whereas UTRs, along with sequences coding the signal peptide (SP), transmembrane (TMD), and cytoplasmic domain (CPD), were from the A/Len/17 HA gene. In addition, the amino acid His at the boundary of the TMD and ectodomain was changed to Tyr since, in a study by Flandorfer et al. [12], the virus with H545Y substitution in chimeric HA on the H1 backbone had significantly improved growth characteristics. The Tyr residue is also in the A/Len/17 HA ectodomain in the corresponding position, so we started the A/Len/17-inherited fragment from this residue.

### 3.2. H2B Virus In Vitro Characteristics

At the first passage after rescue, the engineered virus had a low HA titer (1:4) and was sequentially passaged four times in eggs to improve growth characteristics, followed by cloning by limiting dilutions in eggs. As a result, in the E6 passage virus, the stock titer was 8.3 lgEID_50_/mL at optimal temperature, with an HA titer of 1:64. The virus stock was tested for growth activity at different temperatures in eggs and MDCK cells as well as for its antigenic properties. Full-genome sequencing was performed to reveal possible adaptive mutations that improved virus growth characteristics.

#### 3.2.1. Virus Growth Characteristics, ts/ca Phenotype

The replicative characteristics at different temperatures of the H2B chimera and other viruses used in the study are listed in Table 1. A/Len/17 MDV is a temperature-sensitive (*ts*), cold-adapted (*ca*) virus with an attenuated phenotype [30]. The *ts*/*ca* phenotypes are defined by the viral internal proteins and are inherited by LAIV reassortants prepared on the A/Len/17 backbone [24,31]. Infectious titers of the H2B chimeric virus were similar to that of A/Len/17-based reassortants, with an ability to grow at 26 °C and restricted replication at high temperatures. This is in contrast to the A/Leningrad/134/57 wild-type virus, which grew to a high titer at 39 °C, whereas its replication at low temperature was severely impaired (Table 1). For influenza B viruses, the upper permissive temperature is 36–37 °C due to the features of influenza B biology [32]. For recently circulating influenza B viruses, restricted growth at 38 and 37 °C is common [33]. The H2B chimeric virus differed from the B parent and was able to replicate at 37 and 38 °C, although the titers at these temperatures were low. In MDCK cells, the titer of the chimeric virus was lower than those of the A/Len/17 and B/Brisbane/60/08 strains. Similarly, a lower titer in MDCK cells was observed for the A/LAIV control vaccine (Table 1).

#### 3.2.2. Growth Kinetics in MDCK Cells

**Figure 3:**
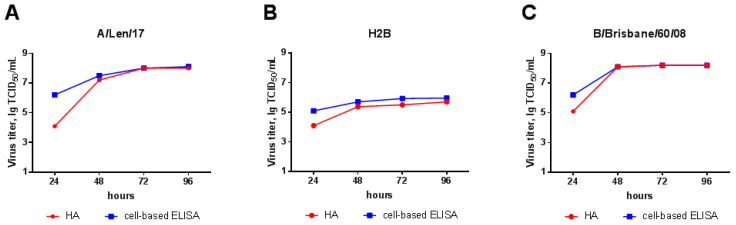
H2B chimera, A/Len/17, and B/Brisbane/60/08 growth kinetics in Madin–Darby Canine Kidney (MDCK) cells: viral replication was detected both by cell-based ELISA with monoclonal antibody to influenza nucleoprotein (NP) and by the HA assay of a cell-culture medium with 1% chicken red blood cells (RBC). (**A**) A/Len/17; (**B**) H2B chimeric LAIV; and (**C**) B/Brisbane/60/2008.

Growth kinetics in MDCK cell culture was studied for the H2B virus and both parental viruses. Virus replication dynamics are shown in Figure 3. The titer of the H2B chimeric virus in the MDCK cell culture was lower than the A/Len/17 and B/Brisbane/60/2008 titers. For all three viruses, detection of intracellular replication with cell-based ELISA at 24 h revealed the virus at higher dilutions than the hemagglutination test, whereas at 48 h and later, the amount of intracellular virus and the virus in the medium became comparable. This effect is common for influenza viruses, and similar results were detected by TCID titration in a study by Lin et al. [34].

#### 3.2.3. HA Thermostability

The thermostability test assesses the stability of the HA trimer molecule. This characteristic positively correlates with the cutoff pH for irreversible HA conformation change, which is associated with a number of important biological characteristics of the influenza virus, such as host range, transmissibility, immunogenicity, and environmental sustainability [35,36,37]. The cutoff temperature of HA inactivation is subtype-specific and is influenced by specific amino acid substitution within the subtype [35,38]. In control LAIVs, the thermostability of HA corresponds to the thermostability of the HA of the epidemic parental strain, a source of the HA gene. For a virus with chimeric HA, this test can give information regarding the functionality of its HA structure. Our results suggest that the chimeric A/B virus has significant HA thermostability: its hemagglutinating activity was retained even after treatment at 60 °C (Table 2), whereas nonstable viruses lose their HA activity after exposure to 52–56 °C [37,39]. Importantly, the thermostability of the H2B chimeric virus was close to that of the B/Brisbane/60/08 virus, the source of the HA ectodomain, and the full-length NA of the A/B chimera. The foreign CPD and TMD of the chimeric HA molecule probably did not dramatically affect the stability of the whole HA protein, indicating that such a construct design is prospective.

#### 3.2.4. Antigenic Characterization of the A/B Chimeric Virus

Antigenic properties of the H2B chimeric virus were assessed by HAI with rat hyperimmune sera raised against a panel of influenza A and B viruses: B/Brisbane/60/08 (B/Victoria), B/Phuket/3073/2013 (B/Yamagata), A/Switzerland/8060/2008 (H3N2), A/LAIV (A/H1N1), B/LAIV (B/Victoria), A/Len/17 (A/H2N2), and B/USSR/60/69. The HAI titers of anti-B/Brisbane/60/08 and anti-B/LAIV sera were identical when tested with the H2B chimeric virus and the corresponding homologic antigen (Table 3), which confirms the antigenic match of H2B HA to B/Brisbane/60/08 HA. The inhibition of hemagglutination by other sera was not detected.

#### 3.2.5. Genetic Stability

**Figure 4:**
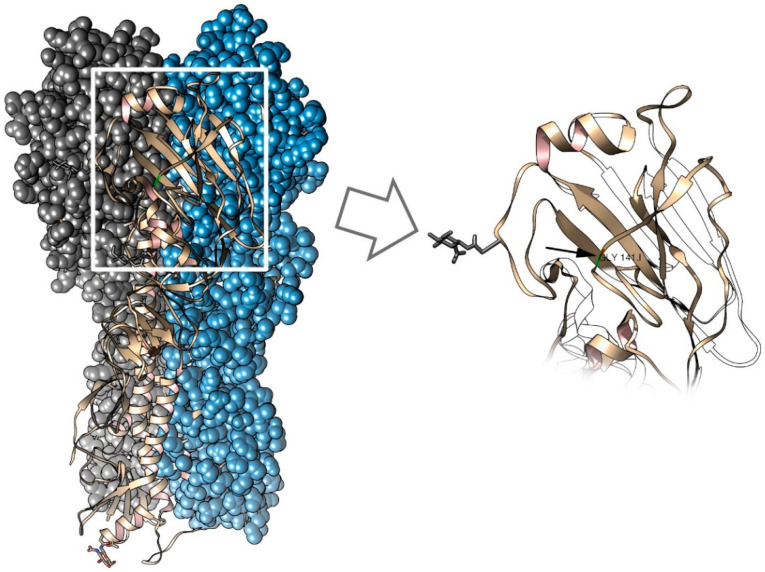
Mapping of the Gly 141 position on the B/Brisbane/60/2008 HA structure. Left: HA trimer, monomers are shown by different colors. Right: the receptor binding site area. The black arrow points to the 141 position. The figure was prepared on the base of PDB 4fqm [42] using Chimera 1.14 software.

During passaging, the growth characteristics of the virus significantly improved from passages E1 to E6. In the E6 stock, the substitution of G to A in the 510 position of the HA gene was found, and this led to the amino acid substitution Gly (GGG) to Glu (GAG) in HA, corresponding to position 141 in HA in B/Victoria numbering. This position is a part of the 150-loop of receptor-binding pocket of the influenza B virus [40], and this change leads to conformational adaptation of the receptor-binding area to another receptor type in eggs [41]. In Figure 4, the 141 position is mapped on the influenza B HA structure.

The sequencing of the full genome revealed additional mutations in the PB1 and NS genes. These mutations did not influence the *ts* phenotype of the chimeric virus but possibly improved growth properties in the eggs. In the PB1 gene, the A to G change in the 706 residue leading to the T228A amino acid change in the PB1 protein and substitution at this position was also found by Jones et al. [43] in A/PR/8/34 PB1 during passaging, in a complex with changes in the PA gene. In the NS gene, 96 G to A mutations led to D24N substitution in the NS1 protein. The 24 amino acid residue is located in the RNA-binding domain and was not mentioned as a key residue for NS1 functions [44]. Salahuddin et al. [45] found that substitution in this position did not influence the functional activity of the RNA-binding domain of NS1.

All attenuating mutations responsible for the *ts* phenotype of A/Len/17 [24] were present in the E10 passage of the H2B chimera, as was established by partial sequencing of the E10 passage virus’s genetic material.

### 3.3. Chimeric H2B LAIV Replication in Mouse Respiratory Tract

**Figure 5:**
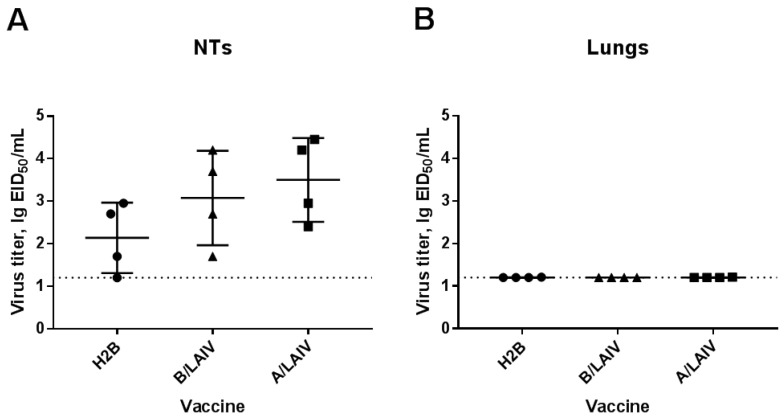
Titers of the studied LAIV viruses in lungs and nasal turbinates (NTs) of C57BL/6J mice on day 3 after immunization (mean ± SD): (**A**) titers in nasal turbinates on day 3 post-immunization and (**B**) titers in lungs at day 3 post-immunization.

Replication of the chimeric A/B virus and the control LAIV viruses in the lungs and nasal turbinates of C57BL/6J mice was studied on day 3 after intranasal immunization. All three viruses were replicated in NTs. Although the titer of the H2B chimeric virus was lower than the titers of control A and B LAIVs, this difference was not significant (Figure 5). In contrast, the titers of all three LAIV viruses in the lungs were under the detection limit, which is indicative of the attenuated phenotype of these viruses. It should be noted that the inoculating dose 10^6^ EID_50_ is only 30 times lower than the viral dose in the licensed LAIV (10^7.5^ EID_50_), and further increasing the dose for testing the vaccine safety was unreasonable because it may result in nonspecific toxicological effects in mice due to excess viral load. Therefore, assessment of the safety of the chimeric A/B vaccine was limited to the dose 10^6^ EID_50_.

### 3.4. Immunogenicity of the Chimeric A/B LAIV

Humoral immune responses of the recombinant virus were assessed three weeks after the second immunization. HAI antibodies, virus-specific serum IgG antibodies, and serum neutralizing activity were measured. Memory T cells targeting an immunodominant H-2^b^-restricted CTL peptide specific for the A/Len/17 virus were studied in spleens five days after influenza A virus challenge.

#### 3.4.1. Antibody Immune Responses

**Figure 6:**
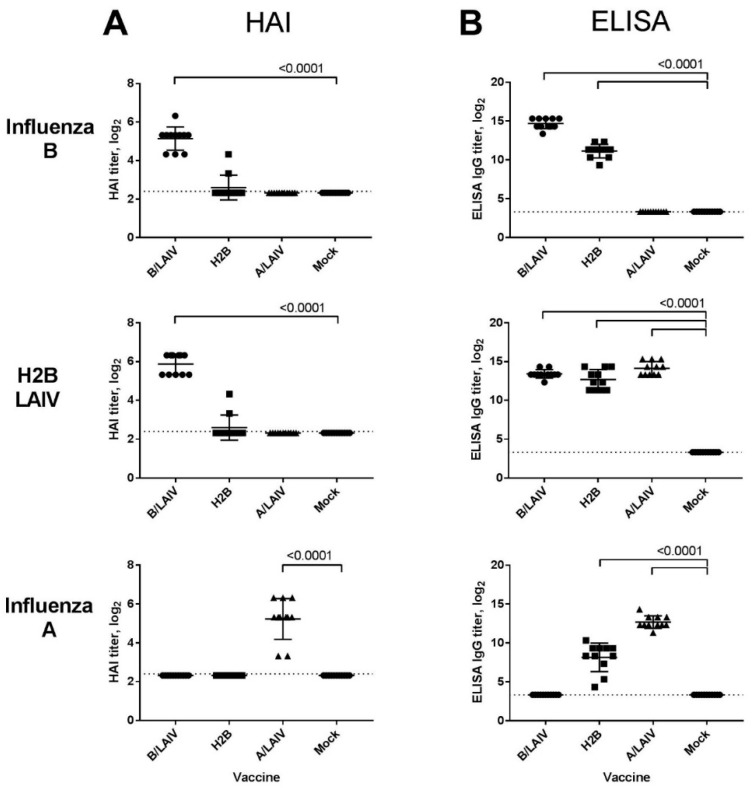
Serum antibody responses after immunization with the LAIVs studied: serum antibody titers were assessed 3 weeks after 2nd immunization. Titers were studied with 3 antigens (influenza B: B/Brisbane/60/2008; H2B LAIV; and influenza A: A/17/New York/2015/5364) in (**A**) hemagglutination inhibition (HAI) and (**B**) ELISA tests. Significant differences are indicated by dashes, *p* values are specified: HAI–Kruskal–Wallis ANOVA (Dunn’s multiple comparisons test); ELISA–ANOVA (Tukey’s multiple comparisons test).

As expected, HAI antibodies in H2B-immunized animals were detected only to the influenza B virus, although the titer was very low: HAI antibodies were detected only in the sera of two animals (Figure 6A). In the control groups, all animals had HAI antibodies to the homologous antigen, and there was no any cross-reactivity between sera of A/LAIV- and B/LAIV-immunized animals.

Total serum IgG against whole virions was measured by ELISA. In the H2B-immunized groups, IgG antibodies against both influenza B and influenza A were detected (Figure 6B). The levels of total IgG to the influenza B antigen were higher than the HAI antibodies and were detected in all animals immunized with a chimeric vaccine. This demonstrates a high level of antibodies that bind to NA or HA parts other than the receptor-binding site. When the A/LAIV whole virus was used as an antigen, this allowed for the detection of antibodies to some internal proteins of A/Len/17 MDV. As was shown in our previous study, A/Len/17-based LAIVs can induce significant levels of serum IgG antibodies targeted at viral NP protein [46]. Importantly, the sera of animals immunized with either B or A/LAIV control vaccines reacted with the H2B antigen at a level identical to the sera of H2B-immunized mice, which further confirms the induction of antibodies to both surface and internal viral proteins after intranasal immunization with LAIVs. As expected, there was no cross-reactivity between the sera of A/LAIV- and B/LAIV-immunized animals (Figure 6A,B).

**Figure 7:**
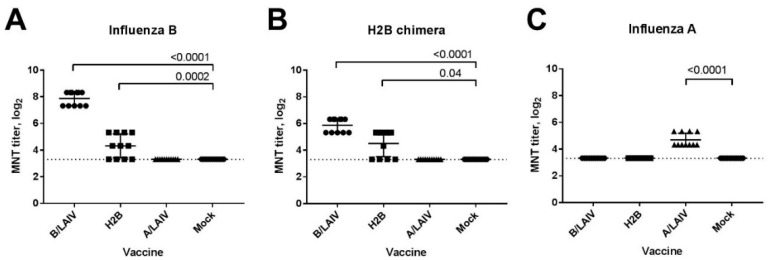
Sera neutralizing activity after immunization with LAIVs studied. Neutralizing antibody titers were assessed in MNT 3 weeks after 2nd immunization, with 3 viruses: (**A**) B/Brisbane/60/2008; (**B**) H2B chimeric LAIV; (**C**) A/17/New York/2015/5364. Significant differences are indicated (Kruskal–Wallis ANOVA, Dunn’s multiple comparisons test).

The sera of H2B-immunized animals have been found to neutralize both the homologous H2B chimeric virus and the natural influenza B virus (Figure 7A,B). Importantly, the MNT titers against these antigens were higher than the corresponding HAI titers, suggesting not only that can antibodies blocking receptor binding neutralize the virus but also that other antibody subsets possess neutralizing activities. Despite the presence of influenza A binding IgG antibodies in the H2B-immunized animals (Figure 6B), there was no neutralization of the influenza A virus by this group (Figure 7C).

#### 3.4.2. T_EM_ Cells in Spleens

**Figure 8:**
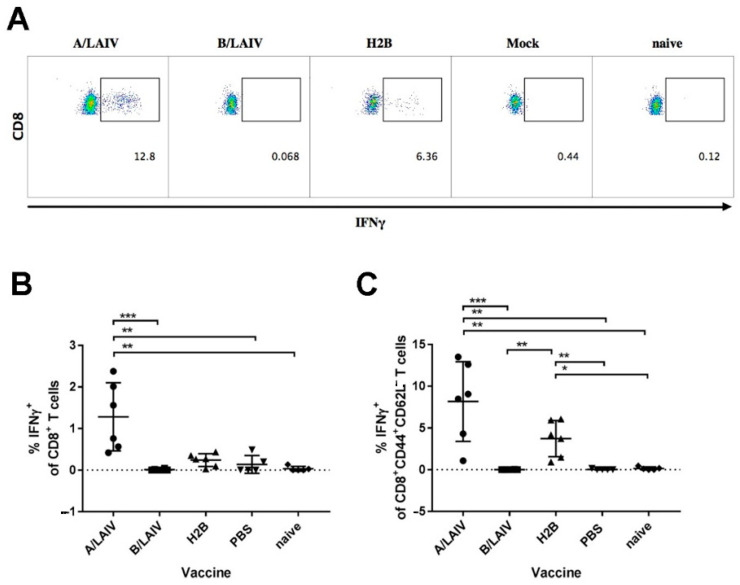
Levels (%) of IFN-γ-secreting CD8^+^ cells and T_EM_ cells (CD8^+^CD44^+^CD62L^−^ phenotype) in spleens of immunized and control mice: splenocytes were isolated 5 days after challenge with influenza A virus, and T-cells were stimulated with influenza A NP_366-374_ CTL peptide (ASNENMDTM). (**A**) Representative flow cytometry plots of the IFNγ-secreting CD8^+^ effector memory T cells for each test group (shown in boxes). The numbers indicate the % of IFNγ-secreting cells among total effector memory CD8^+^ T cells., (**B**) levels of IFNγ-secreting CD8^+^ T cells, and (**C**) levels of IFNγ-secreting CD8^+^ T_EM_ cells (CD8^+^CD44^+^CD62L^−^ phenotype): significant differences are indicated on the graph (Kruskal–Wallis test, multiple comparisons with Dunn’s test); *** *p* < 0.0005; ** *p* < 0.005; and * *p* < 0.05.

For an assessment of T-cell immunity, cytotoxic T cells of effector memory (T_EM_) carrying CD8^+^CD44^+^CD62L^−^ phenotype in spleens, specific to the influenza A immunodominant H-2^b^ peptide NP_366-374_ (ASNENMDTM), were studied with the ICS assay (Figure 8A). Spleens were isolated on the 5th day after challenge with the influenza A virus. Splenocytes were stimulated with the NP_366-374_ peptide and stained with live/dead stain and surface anti-CD4, anti-CD8, anti-CD62L, anti-CD44, and anti-IFN-γ antibodies for the detection of cytokine secreting by T-cell subsets. Interestingly, although the level of total NP_366_-specific CD8 T cells was significantly lower in the H2B-immunized animals compared to the A/LAIV group (Figure 8B), the peptide-specific effector memory subset of these cytotoxic T cells was detected in both A/LAIV- and H2B-immunized animals, and these levels were significantly higher than those in both B/LAIV-immunized and control PBS-immunized groups (Figure 8C). The level of IFN-γ secreted T_EM_ cells in the H2B group was somewhat lower compared to the A/LAIV-immunized group, but this difference was not statistically significant. These data indicate that the chimeric A/B virus is a potent inducer of T-cell memory response to the internal viral protein epitopes, suggesting that the vaccine can protect against influenza A viruses with shared epitopes.

### 3.5. Protective Efficacy of A/B Chimeric LAIV

#### 3.5.1. Protection Against Influenza B Virus

**Figure 9:**
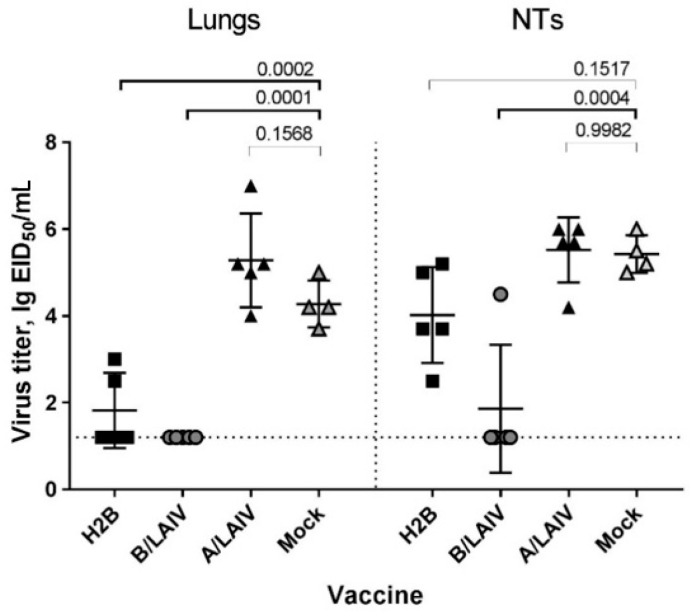
Influenza B challenge virus (B/Brisbane/60/08) titers in lungs and nasal turbinates on 3 dpi: Animals were intranasally (i.n.) inoculated with challenge virus on day 42. Virus titers were studied at 3 dpi by titration in chicken embryos. Titer data are presented as mean ± SD. The *p* values are indicated on the graph (ANOVA, multiple comparisons with Dunnett’s test).

H2B immunization protected mice against the influenza B challenge (B/Brisbane/60/2008). Titers of challenge virus in the lungs were significantly lower than in mock-immunized animals (*p* = 0.0001) (Figure 9). In NTs, a significant viral titer reduction was detected only after immunization with B/LAIV, while in the other groups, viral replication in the upper respiratory tract was not significantly different from the mock-immunized mice based on ANOVA. Immunization with A/LAIV did not protect animals against influenza B, since the titers in the lungs and NTs were not reduced compared to the mock-immunized animals.

#### 3.5.2. Protection Against Influenza A Virus

**Figure 10:**
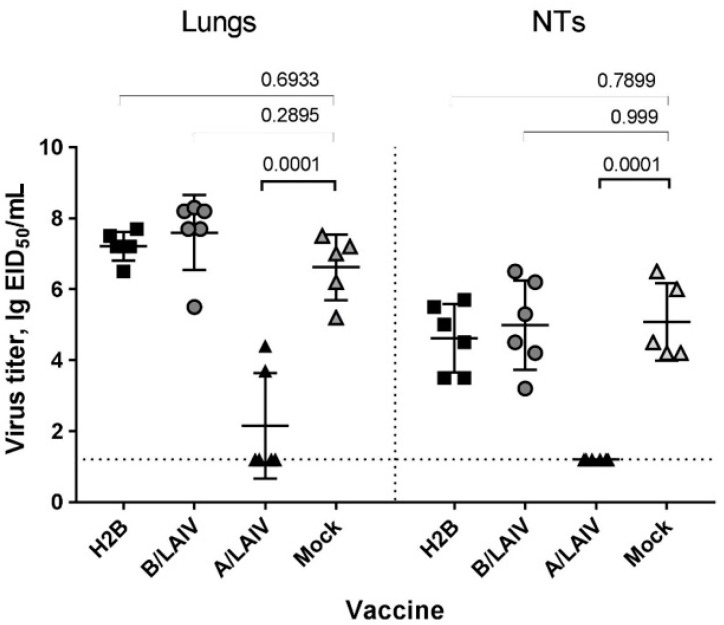
Influenza A challenge virus (A/California/07/2009 mouse-adapted strain) titers in lungs and nasal turbinates on 5 dpi: animals were i.n. inoculated with challenge virus on day 42. Virus titers were studied at 5 dpi by titration in chicken embryos. Titer data are presented as mean ± SD. The *p* values are indicated (ANOVA, multiple comparisons with Dunnett’s test).

In contrast to influenza B challenge, the H2B vaccine did not confer in mice significant protection against virulent influenza A (A/California/07/2009 (H1N1pdm09)) virus infection: the lung and NT titers were comparable to the mock-immunized animals (Figure 10). In addition, there were no significant differences in mouse weight loss between the H2B and control groups. In this experiment, only the A/LAIV vaccine could significantly reduce viral pulmonary titers compared to the control mice, suggesting that antibody responses were the main mediators of protection and that the T cell-based immunity induced by the H2B chimeric virus failed to protect animals against a high dose of mouse-adapted influenza A virus. A possible explanation for this failure is discussed below.

#### 3.5.3. Study of A/California/07/2009 and A/Len/17 Internal Proteins Homology and Epitopes

In similar experiments by Horimoto et al. [47], the protective effect against the backbone virus was demonstrated when the homologous influenza A strain was used for challenge. In our study, we used a heterologous influenza A challenge virus, since homologous H2N2 wild-type viruses matching all epitopes of the internal proteins of the A/Len/17 LAIV backbone have to be handled in the BSL-3 laboratory. The use of a heterologous challenge virus would have demonstrated whether T cell-based immunity to the internal proteins could provide cross-protection by such a chimeric vaccine design. Immunodominant epitopes were described in influenza proteins other than HA and NA; for H-2^b^ mice, the most dominant are NP_366_ and PA_224_ [48], where NP_366_ provides protection and PA_224_ has no protective effect [49]. Both NP_366_ and PA_224_ are different in A/California/07/2009 and A/Len/17 viruses (Table 4). In NP_366_, the differences are located in positions 6 and 7, which potentially make contact with TCR [50]. In influenza A viruses, several variations occur in these positions, and cross-protection against viruses with different amino acid residues in this position was shown to be dose-dependent [51,52]. Nevertheless, immunodominant epitopes play a nonexclusive role in protection; the balance between response to dominant, subdominant responses and protective efficacy depends on the dose and previous influenza exposure [53,54,55]. We compared the sequences of internal proteins of the A/California/07/2009 and A/Len/17 viruses, and the % of differences in these sequences is listed in Table 5. To assess the potential of the H2B vaccine to protect against A/California/07/2009 challenge, we analyzed CTL epitopes experimentally established for H-2^b^ mice, used in our experiments as a model. Information regarding the epitopes was taken from the Immune Epitope Database (IEDB, iedb.org). Analysis of the experimentally described T-cell epitopes for H-2^b^ mice and linear B-cell epitopes for mice from the IEDB demonstrated that only 21% of the established epitopes to date are common among the two viruses, suggesting suboptimal cross-reactivity of the H2B-induced T cells with the A/California/07/2009 heterologous influenza virus (Table 5, Table S1).

Overall, the mouse challenge experiment demonstrated the efficacy of the chimeric A/B LAIV against influenza B virus challenge, whereas efficacy against infection with a heterologous influenza A virus was low, most likely due to the differences in the T-cell epitope content between the vaccine and the challenge viruses. These findings emphasized the necessity for further optimization of epitope composition in such engineered combined vaccine candidates.

## 4. Discussion

The influenza virus’s high variability requires an annual revision of influenza vaccines composition. In a common trivalent formulation, one influenza B strain is included while two strains of different genetic lineages of influenza B are compounds of a quadrivalent formulation. Despite the burden of influenza B in high-risk groups [9,10], in seasons with antigenic mismatch, the rate of influenza B in overall morbidity can reach 70% [9,56], so the role of the influenza B vaccine should not be underestimated. During mixed infection, influenza viruses can interfere, and the impact of this process on live vaccine effectiveness is not fully understood. Influenza B has lower variability and evolution rate [5,57,58]. On the other hand, the suppression of influenza A replication by influenza B NP has been found [59]. The lower variability of influenza B could provide a cross-reactivity of immunity factors and consequently a faster immunity activation after boost with a new vaccine dose; this is discussed as a possible reason for the low vaccine effectiveness of the influenza A component (reviewed in [60]). The ability to prepare strains of both subtypes on the one backbone, in theory, could be a key to solving the interference problem.

Experiments with intertype reassortant rescue have been successfully performed by several groups of scientists [11,12,20,21]. As a backbone, two H1N1 viruses were used: A/WSN/33 [12,20,21] and A/Puerto Rico/8/34 [11]. As a source of influenza B genes, the B/Lee/40 strain was used [20], or B/Yamagata/16/88 [11,12]. However, the aforementioned H1N1 viruses cannot be widely used for the development of human live attenuated vaccines due to the lack of safety data. In our study, the intertype reassortant was rescued on the H2N2 virus backbone with the influenza B strain of B/Victoria lineage, demonstrating that the strategy of the intertype reassortants’ design is universal for influenza viruses of different subtypes. The backbone strain used in this study, A/Len/17, is a master donor virus for a licensed live attenuated influenza vaccine, which has been extensively studied in multiple clinical and epidemiological trials (reviewed in [61]), with established safety, genetic stability, immunogenicity, and effectiveness. In the current study, we performed proof-of-concept experiments with a rescue of an A/Len/17-based A/B reassortant and tested its replication capacity, phenotype, immunogenicity, and ability to protect animals in challenge experiments.

In other studies, A/B chimeric viruses have been shown to have reduced titers and attenuated phenotype in animals [12,20,21]. The H2B chimeric strain on A/Len/17 backbone inherited *ts*, *ca*, and attenuated phenotypes from the vaccine backbone.

The in vitro characteristics of the H2B virus stock were standard for LAIV strains [24,30]. Directly after rescue, the virus had a low titer and low HA activity, but serial passaging in eggs improved its growth characteristics. Sequencing revealed heterogeneity in the virus stock population with an appearance of variants with the substitution G141E in HA specific for influenza B strain egg adaptation [41,62]. The observed changes in PB1 and NS1 could also impact growth characteristics, but mostly important, these substitutions did not alter the *ts* phenotype of the virus and its attenuation in mouse experiment. Nevertheless, the H2B chimeric virus had lower titers in MDCK cells than both parental viruses (A/Len/17 and B/Brisbane/60/08). Of note, the control A/LAIV was also unable to efficient replication in MDCK cells, but still, this vaccine was effective in clinical trials [63], indicating that the virus titer in MDCK is not the main correlate of the vaccine quality. A more significant observation is that the H2B LAIV tends to replicate to lower titers in the upper respiratory tract of mice compared to control vaccines, which could be the reason for lower immunogenicity and decreased protection against influenza A virus. Although these differences were not significant, the tendency for the chimeric vaccine to be less immunogenic than the control B/LAIV could be related to the synergistic effect of the attenuating properties of the backbone and consequences of the viral genetic modification.

In this study, a prime-boost immunization schedule was used to induce optimal immune responses. Although we did not assess antibody and T cell-based immunity after prime immunization, previous studies suggest that a booster LAIV dose indeed significantly enhances adaptive immunity in mice [64,65,66]. The antigenic properties of the constructed chimeric virus assessed in HAI with different sera confirmed that heterologous TMD and CPD in influenza HA did not affect the antigenic properties of the molecule. The sera of H2B-immunized animals reacted with the influenza B antigen in the HAI test and neutralized infectious influenza B virus replication. These data are in line with experiments by Horimoto et al. [22]: after mice infection with an A/WSN/33-based chimeric virus with flu B HA, antibodies to the B antigen in the HAI test were detected. In the current study, HAI and MNT titers after chimeric virus immunization were relatively low, but they were sufficient to protect animals against influenza B virus challenge. It is possible that T-cell responses to HA and NA could have contributed to protection, since serum antibodies are not the only correlate of protection after LAIV immunization [67].

The HA transmembrane domain plays a significant role during fusion, thus affecting the overall stability of the HA molecule [35,68,69]. Low immunogenicity of viruses with unstable HA has been previously reported [36]. During the development of chimeric construction design, we took into consideration the fact that chimeric HAs have to inherit fragments of coding regions from the backbone virus for successful rescue [12,18,19,20]. On the other hand, the stalk region of HA is a significant determinant for cross-reactive antibody response induction [70,71]. Thus, we tested the strategy with TMD and CPD inherited from a backbone virus and other HA domains from a wild-type virus. The possible consequence of inconsistency of the HA transmembrane and stalk domains inherited from viruses of different subtypes could be an unstable HA structure, so we tested this hypothesis in an HA thermostability test. The inhibition of hemagglutination after incubation at high temperatures is an indirect method of assessing HA stability [36,72]. In this test, the chimeric HA has been shown to be stable despite the inconsistency of the ectodomain and transmembrane part of the molecule.

The development of reassortant with influenza B HA and NA on the influenza A backbone gave us a unique opportunity to assess the immunity against influenza A without the impact of influenza A HA and NA proteins in experiments with a live replicating virus. As expected, influenza A neutralizing antibodies and hemagglutination inhibition by the sera of chimera-immunized animals were not detected. IgG antibodies against influenza A were detected by ELISA, and these could be antibody targeted at NP, M1, or M2 proteins. Though antibodies in the internal proteins have no direct virus neutralizing activity, their role in protection, such as NK-cell activation, has been discussed [73]. Importantly, the chimeric vaccine induced significant levels of T_EM_ cells (CD44^+^CD62L^−^ phenotype) in spleens specific to A/Len/17 NP_366-374_ peptide. Although we did not assess T-cell immunity in the whole influenza A virus, stimulation of the splenocytes with the most immunodominant epitope reflects the overall cell-mediated immune responses to vaccination, and stimulation with the whole virus yields similar results in terms of intergroup comparison [52].

In Horimoto et al.’s experiments [47] with A/B chimeras, the protective response to the backbone influenza A virus was shown. In these chimeras, only HA was transferred to the chimeric virus. NA was from the backbone virus. In addition, the backbone that was used to prepare the chimeric viruses was not attenuated, and the same virus was used for challenge. In our study, we used more relevant influenza A backbone for the construction of a chimeric vaccine, a LAIV master donor virus of H2N2 subtype, which is routinely used for preparation of seasonal influenza vaccines against circulating H1N1 and H3N2 influenza strains. Here, we were unable to challenge mice with the original H2N2 1957 pandemic virus due to safety reasons. However, influenza viruses share common T-cell epitopes that, in theory, could provide partial protection against infection with drifted influenza viruses [74]. Therefore, we selected a recent heterologous influenza A/H1N1 to assess protective effect of the chimeric vaccine against circulating strains. Despite effective T-cell immunity stimulation by the H2B chimeric vaccine, it was not sufficient to protect animals against a high-dose influenza A virus of the heterologous subtype. It is of special note that the most immunodominant epitopes of influenza A virus internal proteins have significantly evolved since 1957 (the year of A/Len/17 strain isolation) and are not present in the currently circulating viruses anymore. Furthermore, the differences even in one immunodominant epitope, such as NP_366_, can significantly alter the protective efficacy of the LAIVs in C57BL/6J mice [46]. Indeed, matching of the internal proteins’ sequences revealed that a high proportion of H-2^b^-restricted T-cell epitopes of A/Len/17 virus differ from that of A/California/07/09 virus, so the protective role of T-cell immunity formed after immunization in C57BL/6J mice could be decreased.

We discussed earlier the issue of mismatch between the T-cell epitope composition in reassortant viruses for live and inactivated influenza vaccines and currently the circulating influenza viruses and made recommendations reconsidering the genome composition of the vaccines by the replacement of either NP or M genes of the old attenuated backbone virus with that of a recent influenza strain, which would enhance the cross-protective properties of the vaccines in terms of optimizing T-cell immunity [75]. Our current study further emphasized the necessity for optimizing genome composition of the chimeric vaccine construct to enhance the cross-reactivity of T-cell responses against influenza A viruses. Further experiments with optimized construction designs, vaccine dosages, and immunization schedules are warranted, as they could improve immunogenicity and the protective potential of the chimeric vaccine.

## 5. Conclusions

A chimeric A/B LAIV reassortant based on the H2N2 cold-adapted backbone was successfully rescued as a proof-of-concept combined vaccine prototype. In vitro characteristics of the chimeric virus correlated with that of the classical 6:2 LAIV reassortant (the temperature-sensitive phenotype, attenuation for animals, and antigenicity matching with HA parent). Despite reduced antibody titers against the influenza B virus, the vaccine protected mice against the influenza B challenge. The vaccine efficiently induced memory T-cell responses to the backbone internal protein epitopes, but these responses were not sufficient for protection against the heterologous influenza A virus, and optimization of epitope composition in the backbone virus is needed to overcome this issue.

## Figures and Tables

**Table 1:** Growth characteristics of studied viruses at different temperatures.

Virus ^1^	Virus Reproduction at Different Temperatures					
	lg EID_50_/mL					lg TCID_50_/mL
	26 °C	33 °C	37 °C	38 °C	39 °C	33 °C
H2B	5.0	8.3	3.2	2.2	<1.2	5.9
A/Len/17	6.5	9.0	6.9	3.9	<1.2	8.0
B/Brisbane/60/08	2.2	8.7	<1.2	<1.2	<1.2	8.2
A/Leningrad/134/57	3.1	8.5	8.5	8.4	8.0	8.9
A/LAIV (NY)	5.0	7.5	3.8	1.5	<1.2	5.3
B/LAIV (Bris)	5.7	8.5	<1.2	<1.2	<1.2	7.6

^1^ H2B: A/B chimeric reassortant LAIV; A/Len/17: A/Leningrad/134/17/57; A/LAIV (NY): A/17/New York/2015/5364; B/LAIV: B/60/Brisbane/2008/83.

**Table 2:** HA thermostability test results for the H2B chimeric virus and other viruses under the study.

Virus	Reciprocal HA Titer After Incubation at Indicated Temperatures							
	Non-Heated	37 °C	50 °C	54 °C	56 °C	58 °C	60 °C	65 °C
H2B	64	64	64	64	64	64	16	0
A/Len/17	256	128	128	128	128	128	128	0
B/Bris/60	32	32	32	32	32	32	16–32	0
A/LAIV (NY)	256	256	128	64	32	32	16	0
B/LAIV (Bris)	64	64	64	64	64	64	64	0

**Table 3:** Antigenic properties of the H2B chimeric virus.

Hyperimmune Sera		HAI Titer with H2B Virus Antigen
Raised Against Virus	HAI Titer with Homologous Antigen	
B/Brisbane/60/08 (B/Victoria)	320	320
B/Phuket/3073/2013 (B/Yamagata)	640	<10
A/Switzerland/8060/2008 (A/H3N2)	640	<10
A/LAIV (A/H1N1)	320	<10
B/LAIV (B/Victoria)	640	640
A/Len/17 (A/H2N2)	1280	<10
B/USSR/60/69	640	<10

**Table 4:** Immunodominant epitopes for H-2^b^ haplotype mice NP_366_ and PA_224_ in A/Len/17 and A/California/07/2009 proteins.

Epitope	NP_366_	IEDB ID	PA_224_	IEDB ID
**A/Len/17**	ASNENMDTM	4580	SCLENFRAYV	57105
**A/California/07/2009**	ASNENVETM	4630	PSLENFRAYV	175633

**Table 5:** Difference of the internal proteins of the A/Len/17 and A/California/07/2009 viruses and experimentally established epitopes for mice according to the Immune Epitope Database (IEDB).

Protein	Hamming Distance, % (Different Amino Acids/Protein Length)	Epitopes for H-2^b^ Mice ^1^	Linear B Epitopes for Mice ^2^	Total
		Common ^3^/All	Common/All	
PB2	4% (34/759)	12/29	-	12
PB1	4% (28/757)	16/26	-	16
PA	5% (37/716)	20/34	-	20
NP	9% (43/498)	16/98	3/12	19
M1	6% (15/252)	3/13	2/8	5
M2	15% (15/97)	0/3	2/76	2
NS1	18% (40/219 (237))	1/12	2/8	3
NEP	12% (15/121)	1/7	-	1

^1^ Experimentally established as positive epitopes of the influenza A virus (organism ID 11320) selected from IEDB for the H-2^b^ haplotype mice. ^2^ Experimentally established as positive in B-cell assays of the linear epitopes of the influenza A virus (organism ID 11320) selected from IEDB for mice. ^3^ Common epitopes: identical in A/Len/17 proteins and A/California/07/2009. All: deposited in IEDB for influenza A viruses (all strains) with limitations specified in ^1,2^.

## Data Availability

The data presented in this study are available on request from the corresponding author.

## Supplementary Materials

The following are available online at https://www.mdpi.com/2076-2607/9/2/259/s1: Table S1: Common epitopes for mice in proteins of A/Len/17 and A/California/07/2009 influenza virus strains.

## Glossary

AUC	area under the curve
ANOVA	analysis of variance
BSA	bovine serum albumin
*ca*	cold-adapted
CTL	cytotoxic T lymphocyte
CPD	cytoplasmic domain
DMEM	Dulbecco’s Modified Eagle Medium
EID_50_	50% egg infective dose
ELISA	enzyme-linked immunosorbent assay
FBS	fetal bovine serum
HAI	hemagglutination inhibition assay
H2B	chimeric A/B LAIV strain
HRP	horseradish peroxidase
IEDB	immune epitope database
IFNγ	interferon gamma
ICS	intracellular cytokine staining
i.n.	intranasal
LAIV	live attenuated influenza vaccine
A/Len/17	A/Leningrad/134/17/57 (H2N2) virus
MHC	major histocompatibility complex
MDCK	Madin–Darby Canine Kidney cell line
MDV	master donor virus
MNT	microneutralization test
NA	neuraminidase
NT	nasal turbinates
OD	optical density
PBS	phosphate-buffered saline
PCR	polymerase chain reaction
RBC	red blood cells
SD	standard deviation
SP	signal peptide
TCID_50_	50% tissue infective dose
TMD	transmembrane domain
T_EM_	effector memory T cells
*ts*	temperature sensitive
UTR	untranslated region
