# Supplementary material for: Safety, Immunogenicity, and Protective Efficacy of a Chimeric A/B Live Attenuated Influenza Vaccine in a Mouse Model
Source: Microorganisms. 2021 Jan 27;9(2):259. doi: 10.3390/microorganisms9020259 (PMC7910998; doi:10.3390/microorganisms9020259)
Supplement: Supplementary file 1 [file microorganisms-09-00259-s001.pdf]

Supplementary Table 1. Common experimentally established epitopes for C57BL/6J mice in proteins of A/Len/17 and A/California/07/2009 influenza virus strains.

PB2	PB1	PA	NP	M1	M2	NS1	NEP
68849	62904	10910	26315	37043 (B)[1]	97650 (B)	178131 (B)	36279
54437	62905	30549	18406	129610 (B)	141748 (B)	509227 (B)	
72047	18326	153638	175601	178133		36801	
36552	31094	20399	67436	41602			
23329	42121	12696	65994	28309			
58476	60934	175570	49219				
29179	70049	53218	41732				
62239	15947	130369	5757				
49712	19640	23219	17539(B)				
65229	129308	72054	53639 (B)				
70836	44946	3070	128638				
69735	36383	55634	55796				
	15880	33793	194962				
	61497	58385	182545				
	130036	10777	129242				
	53812	34291	939005				
		60765	175669				
		2290	15381 (B)				
		59587	39214				
		36128					

[1] linear epitopes, established in B-cell assays. The epitopes were selected from Immune Epitope Database (iedb.org), in the table IEDB IDs are indicated. From all deposited in IEDB, linear peptides were selected, established for influenza A viruses (organism ID 11320), specific for H-2[b] haplotype mice; and all linear epitopes for mice, established by B-cell assays. To select common epitopes, alignment of A/Len/17 and A/California/07/2009 proteins was performed. Epitopes, identical in A/Len/17 and A/California/07/2009 proteins were considered as common.
